# Supplementary material for: Modulation of the Promoter Activation Rate Dictates the Transcriptional Response to Graded BMP Signaling Levels in the Drosophila Embryo
Source: Dev Cell. 2020 Sep 28;54(6):727–741.e7. doi: 10.1016/j.devcel.2020.07.007 (PMC7527239; doi:10.1016/j.devcel.2020.07.007)
Supplement: Document S1. Figures S1–S7 and Table S1 [file mmc1.pdf]

Developmental Cell, Volume *54*

Supplemental Information

Modulation of the Promoter Activation Rate Dictates the Transcriptional Response to Graded BMP Signaling Levels in the *Drosophila* Embryo

Caroline Hoppe, Jonathan R. Bowles, Thomas G. Minchington, Catherine Sutcliffe, Priyanka Upadhyai, Magnus Rattray, and Hilary L. Ashe

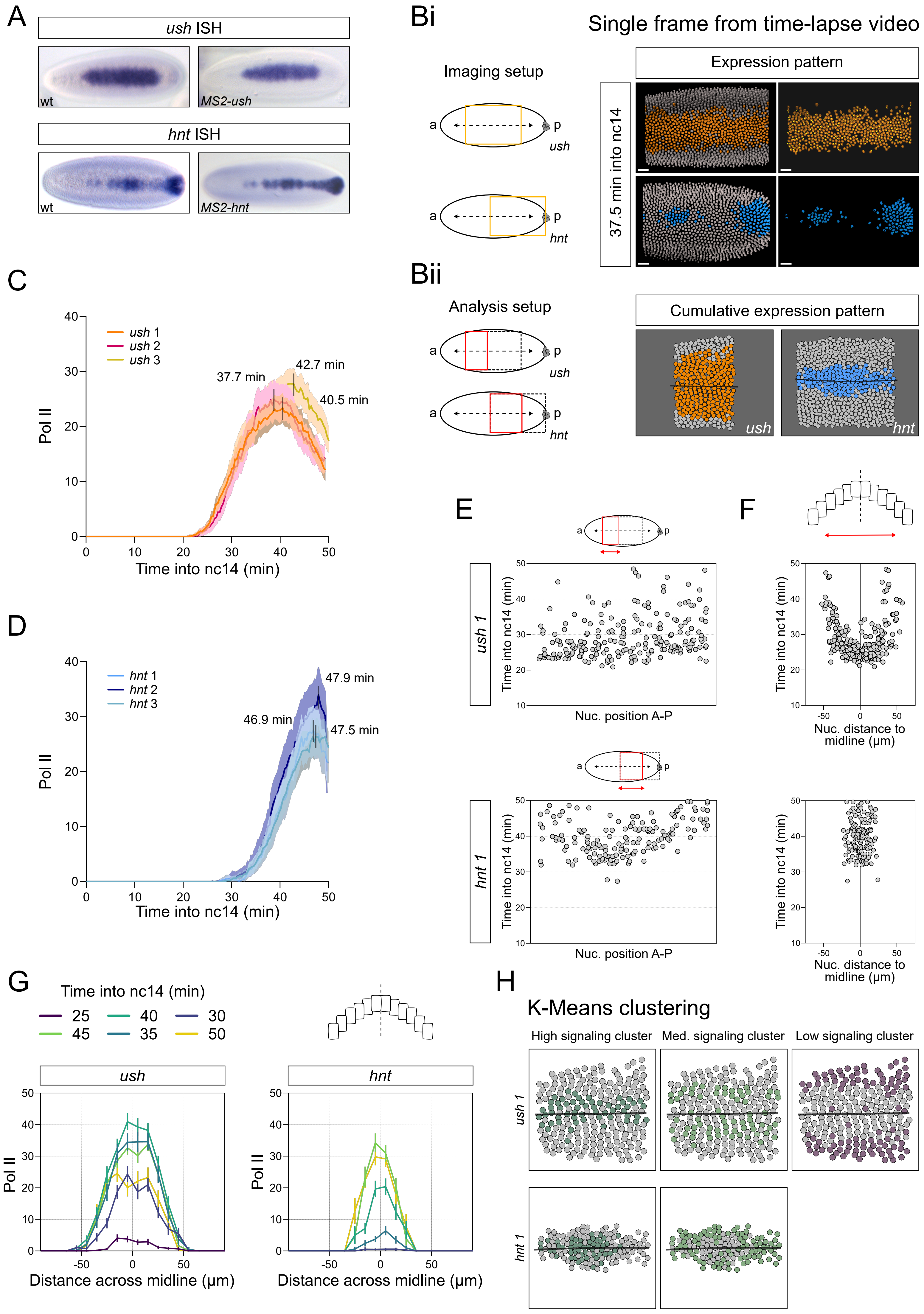

A
ush ISH
wt
MS2-ush
hnt ISH
wt
MS2-hnt
Bi
Single frame from time-lapse video
Imaging setup
a
p
ush
hnt
Expression pattern
37.5 min into nc14
Bii
Analysis setup
Cumulative expression pattern
C
Pol II
Time into nc14 (min)
ush 1
ush 2
ush 3
37.7 min
42.7 min
40.5 min
D
hnt 1
hnt 2
hnt 3
47.9 min
46.9 min
47.5 min
E
ush 1
hnt 1
Time into nc14 (min)
Nuc. position A-P
F
Nuc. distance to midline (μm)
G
Time into nc14 (min)
25
40
30
45
35
50
Distance across midline (μm)
H
K-Means clustering
High signaling cluster
Med. signaling cluster
Low signaling cluster

SUPPLEMENTARY FIGURE LEGENDS

Figure S1: Characterization of *ush* and *hnt* transcription dynamics using live imaging. Related to Figure 1.

(A) *In situ* hybridization images of *ush* (top) and *hnt* (bottom) in WT embryos and embryos homozygous for the MS2 tagged gene locus.

(Bi) Schematics of the *ush* and *hnt* imaging domains (yellow boxes). Stills from time-lapse data sets (Video S1, S2) false colored for active transcription show the expression pattern at 37.5 min into nc14.

(Bii) Schematics show the *ush* and *hnt* analysis domains (red boxes) and the cumulative expression patterns for representative embryos.

(C, D) Mean transcription traces shown for *ush* (C) and *hnt* (D) over time, with the time taken to reach the maximum Pol II number shown for all biological replicates (n = 3 for *ush* 209, 186, 223 nuclei and *hnt* 144, 171, 229 nuclei).

(E, F) The transcription onset time of *ush* and *hnt* is plotted according to the nuclear position along the AP (E) and DV (F) axes for one representative biological replicate. Each dot represents one nucleus.

(G) Mean number of Pol II transcribing *ush* and *hnt* divided into positional bins along the embryo cross section. Bin width = 10 μm, time resolution = 5 min. Data were pooled from all biological replicates (n= 618 *ush*, 544 *hnt* nuclei).

(H) K-Means clustering analysis identified three clusters for *ush* and two for *hnt*. Expression domains of representative embryos are false colored according to their cluster.

Scale bar, 20μm (Bi). Mean ± 95% confidence intervals (C, D, G).

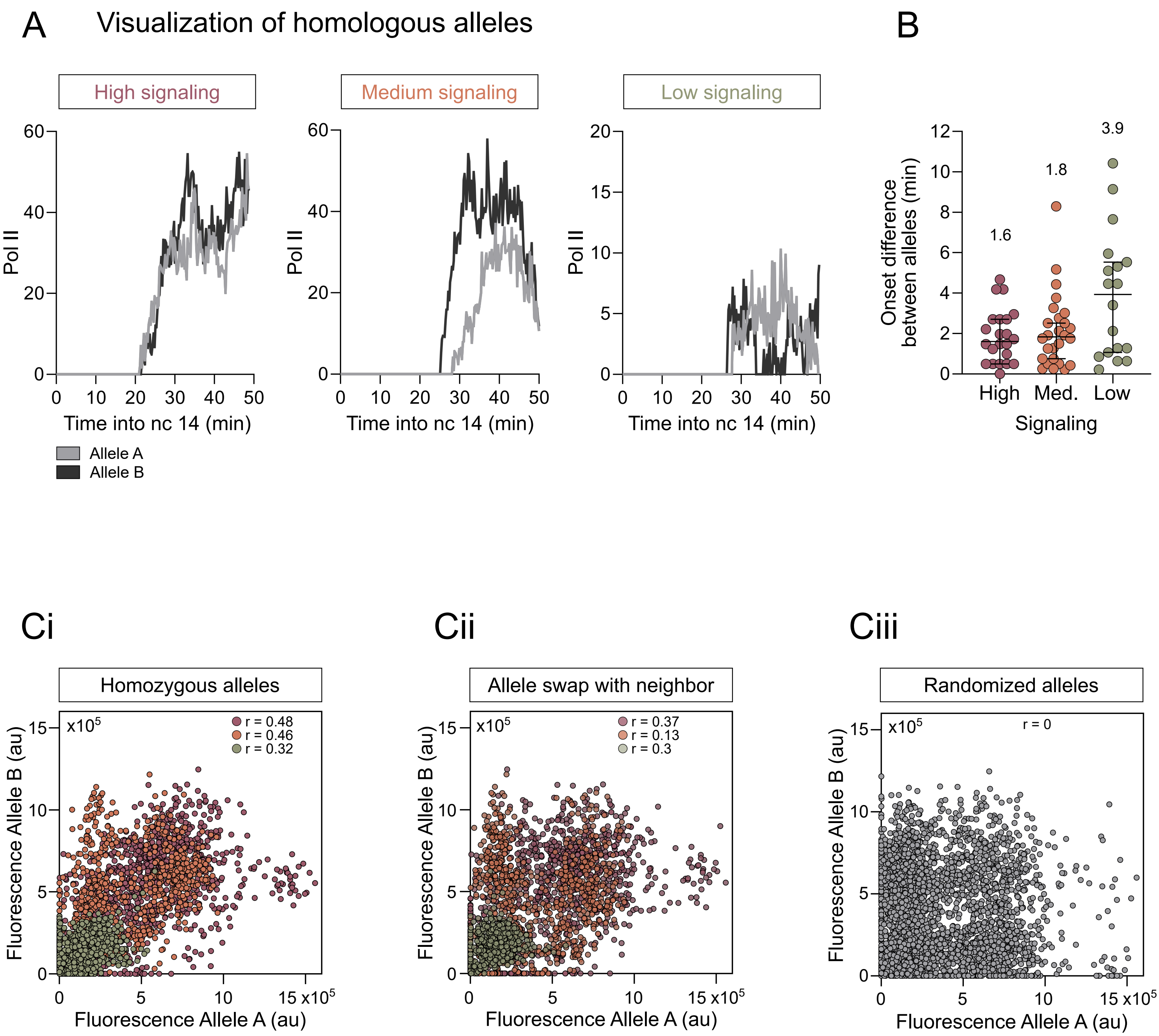

A
Visualization of homologous alleles
High signaling
Medium signaling
Low signaling
Pol II
Time into nc 14 (min)
Allele A
Allele B
B
Onset difference between alleles (min)
1.6
1.8
3.9
High
Med.
Low
Signaling
Ci
Homozygous alleles
x10^5
r = 0.48
r = 0.46
r = 0.32
Cii
Allele swap with neighbor
r = 0.37
r = 0.13
r = 0.3
Ciii
Randomized alleles
r = 0
Fluorescence Allele B (au)
Fluorescence Allele A (au)

Figure S2: Analysis of transcription from homozygous *ush*-MS2 alleles. Related to Figure 1.

(A) Representative *ush* transcription traces from embryos homozygous for the *ush*-MS2 imaging locus according to the nuclear position in the BMP signaling gradient experiencing high, medium or low signaling. Alleles were assigned randomly. Note different y-axis scales.

(B) Differences in transcription onset time between homologous alleles. Median difference in minutes is given above data.

(C) Fluorescence intensities of individual alleles at any time point plotted with their partner in the same nucleus (i) or following exchange with an allele in the closest neighboring nucleus (ii) or after a completely random exchange (iii). Alleles are colored depending on the spatial position of the nucleus.

Median ± 95% confidence intervals (B). Pearson correlation coefficient is shown for each pair of variables between fluorescence of allele A and allele B (Ci-Ciii).

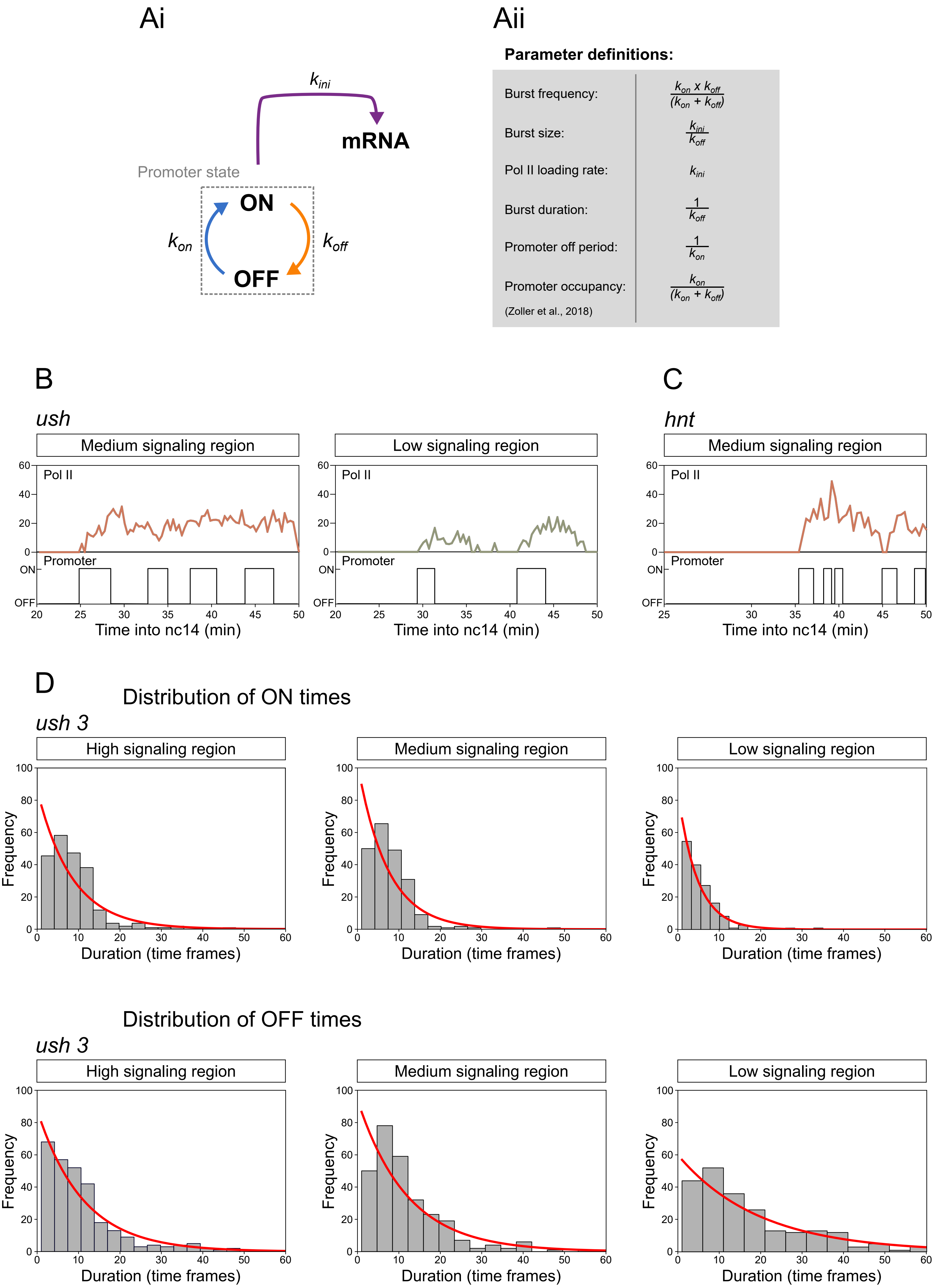

Ai
Aii
Parameter definitions:
Burst frequency: $\frac{k_{on} \times k_{off}}{(k_{on} + k_{off})}$
Burst size: $\frac{k_{ini}}{k_{off}}$
Pol II loading rate: k_{ini}
Burst duration: $\frac{1}{k_{off}}$
Promoter off period: $\frac{1}{k_{on}}$
Promoter occupancy: $\frac{k_{on}}{(k_{on} + k_{off})}$
(Zoller et al., 2018)
k_{ini}
mRNA
Promoter state
ON
OFF
k_{on}
k_{off}
B
C
ush
hnt
Medium signaling region
Low signaling region
Pol II
Promoter
Time into nc14 (min)
D
Distribution of ON times
Distribution of OFF times
ush 3
High signaling region
Frequency
Duration (time frames)

Figure S3: Inference of *ush* and *hnt* promoter states. Related to Figure 2.

(A) Schematic of a two-state promoter model where the promoter switches between an active ON and an inactive OFF state. When in the ON state mRNA is produced with the rate of k_{ini}. The probability of switching between the two states is described by the rates k_{on} and k_{off} (i). Burst parameter definitions (Zoller et al. 2018) used to investigate changes in burst kinetics (ii).

(B, C) Representative transcription traces and inferred promoter states for *ush* (B) and *hnt* (C) from nuclei receiving medium and low levels of BMP signaling.

(D) Graphs show histograms of the distribution of promoter ON and OFF periods for the high, medium and low signaling regions of *ush* WT embryo 3, along with a corresponding geometric distribution derived from the HMM transition matrix for each region.

A

	Occup.	k_{on}	k_{off}	Load. rate	Freq.
ush wt 1	0.99	0.70	-0.45	0.11	0.31
ush wt 2	0.91	0.84	-0.46	0.45	0.63
ush wt 3	0.99	0.84	-0.62	0.07	0.53

B

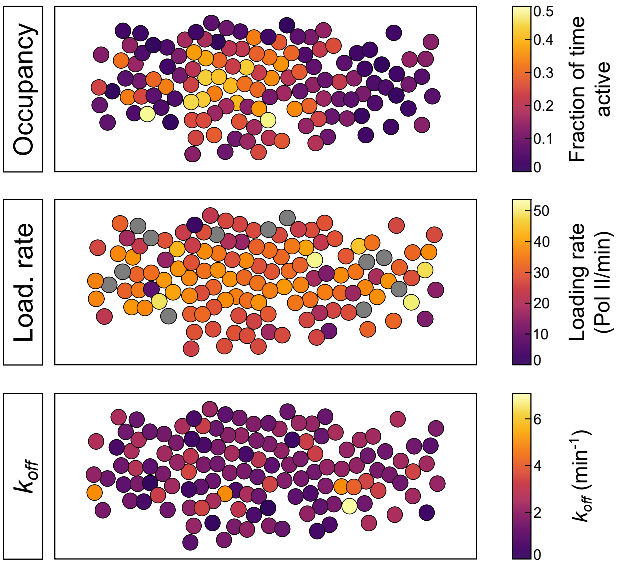

C

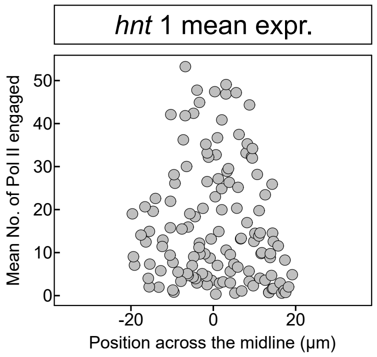

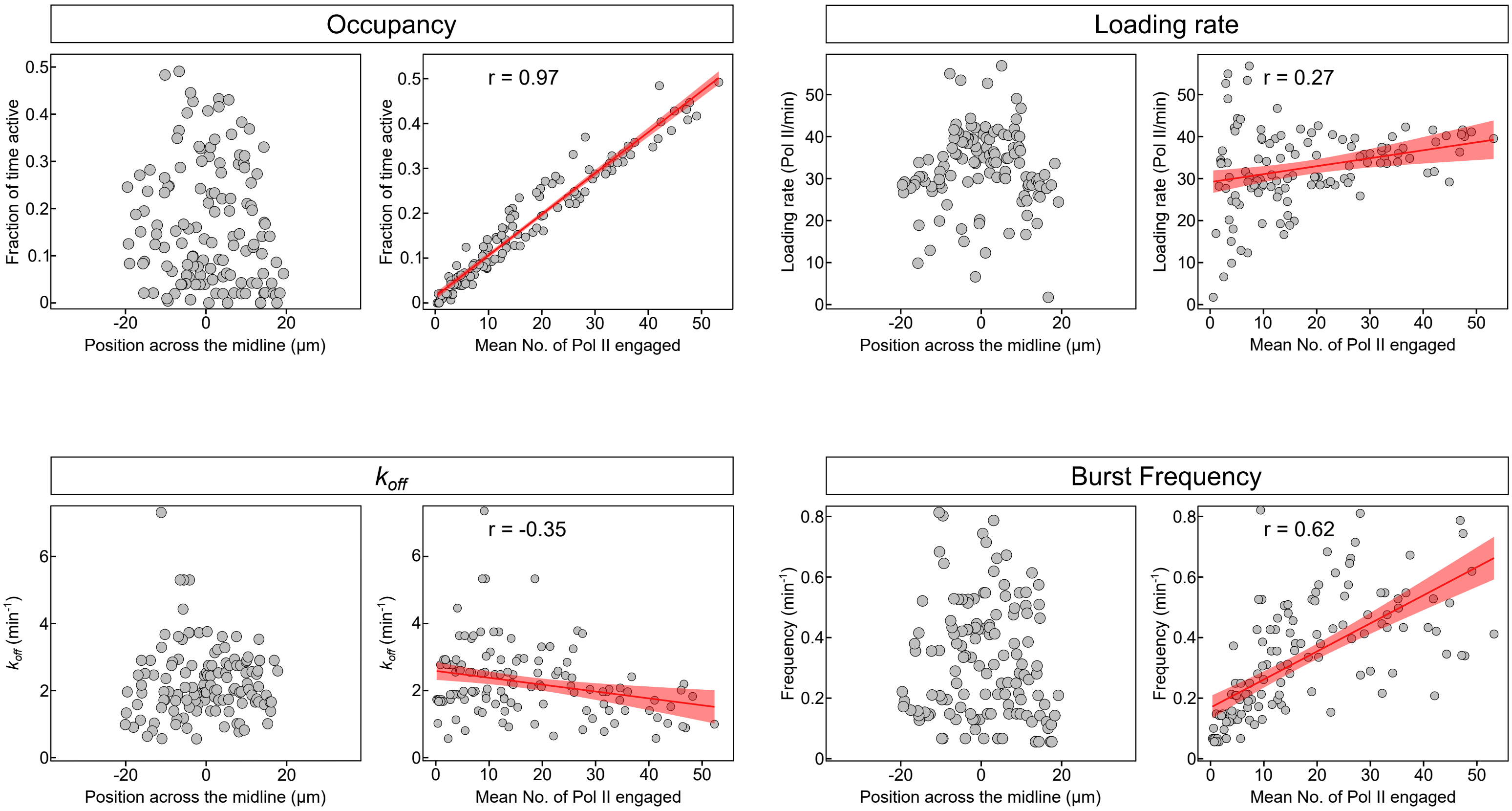

D

	Occup.	k_{on}	k_{off}	Load. rate	Freq.
hnt wt 1	0.97	0.74	-0.35	0.27	0.62
hnt wt 2	0.99	0.72	-0.45	0.28	0.57
hnt wt 3	0.99	0.67	-0.48	0.41	0.46

Figure S4: Single cell burst parameters in response to different BMP signaling levels. Related to Figure 3.

(A) Pearson correlation coefficients shown for each pair of variables between *ush* burst parameter and mean expression for all biological replicates.

(B) *hnt* expression domain of a representative embryo shown as heatmaps with nuclei false colored according to their single cell transcription parameters for occupancy, loading rate and k_{off}. Gray colored nuclei in the loading rate heatmap represent nuclei where the single cell loading rate parameter was not defined, due to the fitted model outputting an empty promoter trace. Scales as indicated.

(C) Mean expression of nuclei transcribing *hnt* plotted according to nuclear position across the dorsal midline for one representative embryo. Promoter occupancy, loading rate, k_{off} and burst frequency of single *hnt* nuclei plotted based on position (left) and against mean expression (right).

(D) Pearson correlation coefficients shown for all biological replicates of *hnt* between burst parameter and mean expression.

Linear regression ± 95% confidence intervals and Pearson correlation coefficient is shown, n= 131 nuclei (119 for loading rate) (B, C).

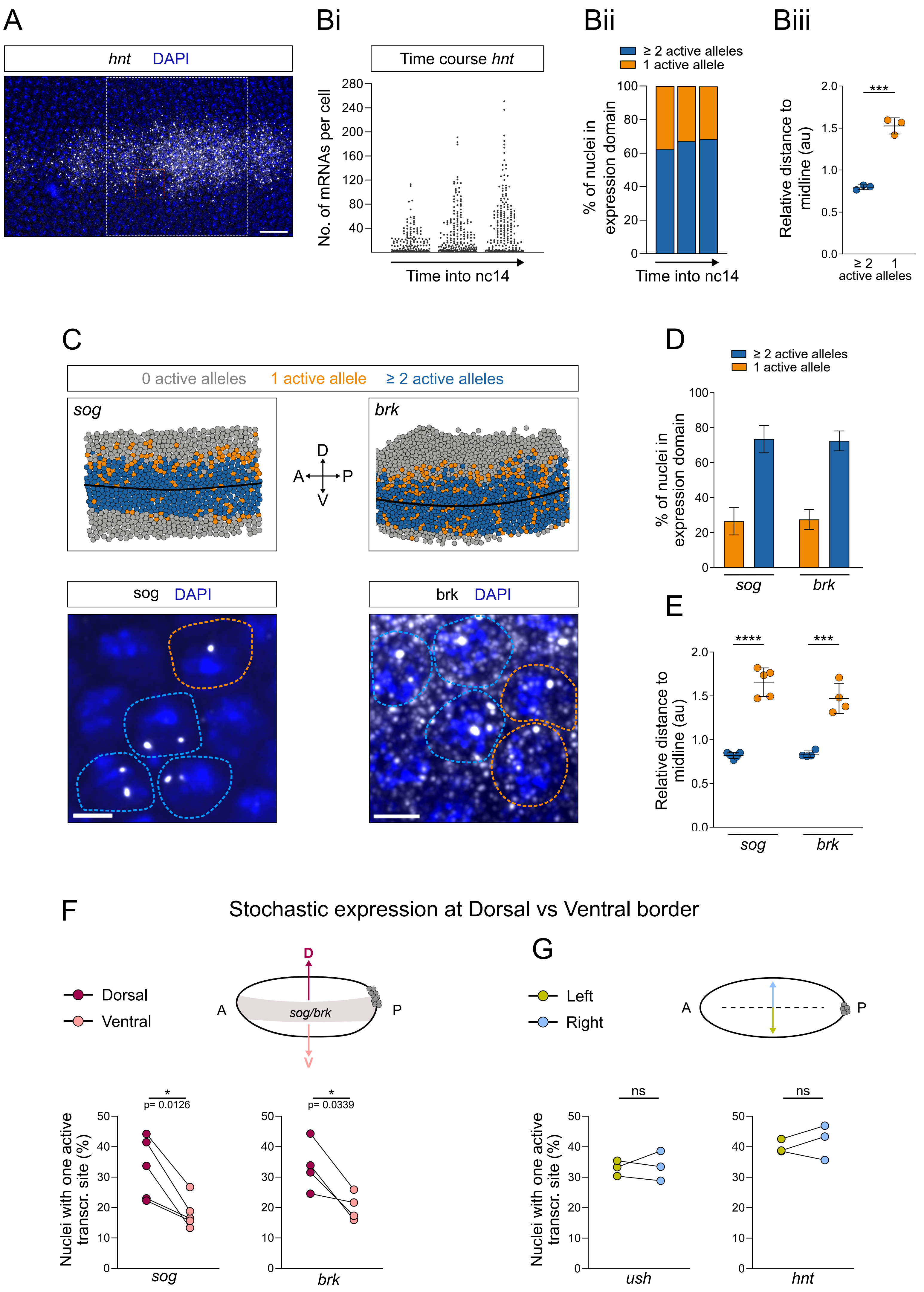

A
hnt DAPI
Bi
Time course hnt
No. of mRNAs per cell
Time into nc14
Bii
≥ 2 active alleles
1 active allele
% of nuclei in expression domain
Time into nc14
Biii
Relative distance to midline (au)
***
≥ 2
1
active alleles
C
0 active alleles
1 active allele
≥ 2 active alleles
sog
brk
D
A
P
V
sog DAPI
brk DAPI
D
≥ 2 active alleles
1 active allele
% of nuclei in expression domain
sog
brk
E
Relative distance to midline (au)
****
***
sog
brk
F
Stochastic expression at Dorsal vs Ventral border
Dorsal
Ventral
A
P
sog/brk
D
V
Nuclei with one active transcr. site (%)
*
p= 0.0126
sog
*
p= 0.0339
brk
G
Left
Right
A
P
ns
ush
ns
hnt

Figure S5: BMP target gene mRNA output and stochastic transcription of Dorsal target genes. Related to Figure 4.

(A) Expression pattern of *hnt* visualized with exonic smFISH probes. Region outlined by the red box is shown in the top panel of Figure 4E and the white box is shown in the schematic in Figure 4E.

(B) Time course of *hnt* mRNA number per cell in embryos of increasing age in nc14 (i), their percentage of nuclei with one (orange) or two (blue) active alleles (ii) and their median distance to the middle of the expression domain (iii).

(C) Representation of the expression patterns of Dorsal target genes *sog* and *brk* in *Drosophila* embryos (lateral views), with nuclei false colored according to the number of active transcription foci based on FISH images (not shown). A line of best fit shows the middle of the expression domain. Enlarged regions from FISH images (lower panels) showing nuclei with one (orange outline) or two (blue outline) active transcription sites.

(D) Proportion of nuclei with one or two nascent transcription sites within the expression domain.

(E) Distance to the middle of the expression domain is plotted for nuclei with one or two transcriptionally active alleles.

(F) Proportion of nuclei with one active transcription site in the dorsal (purple) versus the ventral (pink) half of the expression domain for each embryo analyzed in (D).

(G) Analysis of nuclei identified with one active transcription site of the BMP target genes *ush* and *hnt* between each half (green or blue) of the expression domain. Data for BMP target genes is from FISH images (not shown).

Embryos oriented dorsally (A) and laterally (C) with anterior to the left. Biological replicates = 3 (*hnt*), 5 (*sog*) and 4 (*brk*). Scale bar, 25 μm (A) and 3 μm (C). *$p < 0.05$, ***$p < 0.001$, ****$p < 0.0001$; ns, not significant. Mean ± SD (Biii, D, E). Student's t-test (Biii, E) or a paired Student's t-test (F, G).

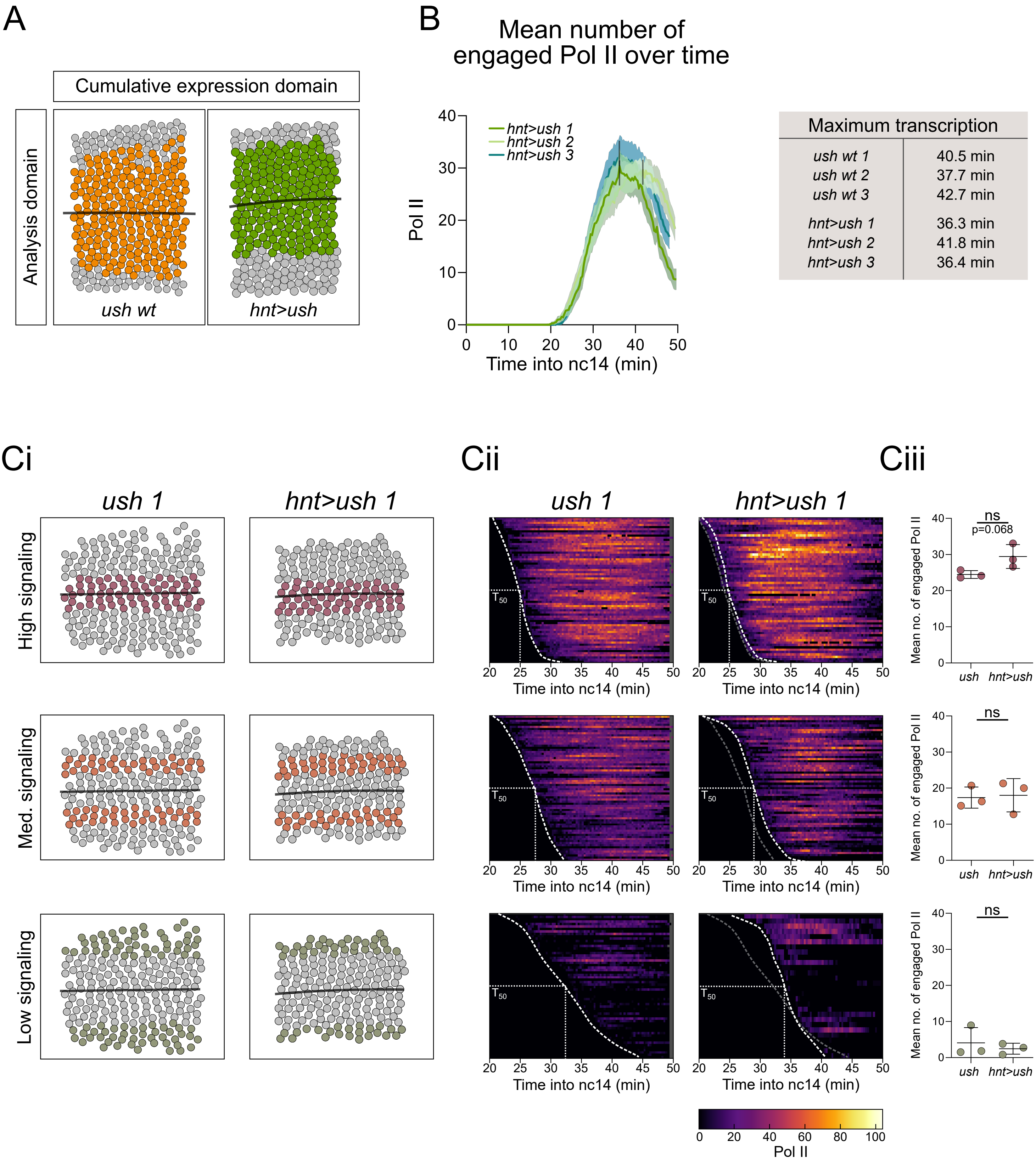

A
Cumulative expression domain
Analysis domain
ush wt
hnt>ush
B
Mean number of engaged Pol II over time
hnt>ush 1
hnt>ush 2
hnt>ush 3
Pol II
Time into nc14 (min)
Maximum transcription
ush wt 1 40.5 min
ush wt 2 37.7 min
ush wt 3 42.7 min
hnt>ush 1 36.3 min
hnt>ush 2 41.8 min
hnt>ush 3 36.4 min
Ci
Cii
Ciii
ush 1
hnt>ush 1
High signaling
Med. signaling
Low signaling
T50
Time into nc14 (min)
Mean no. of engaged Pol II
ns
p=0.068
ush
hnt>ush
Pol II

Figure S6: Analysis of *hnt>ush* embryos. Related to Figure 6.

(A) Cumulative expression domains of *ush* transcription in *ush* (orange) and *hnt>ush* (green) embryos.

(B) Mean *ush* transcription in *hnt>ush* embryos, with time of maximum transcription detected listed for all replicates and *ush* WT embryos for comparison (n = 3, 154, 187, 202 nuclei).

(Ci) The regions that were analyzed, based on BMP signaling levels, are shown by false coloring for one representative embryo per genotype.

(Cii) Heatmaps of single-cell traces, sorted according to transcription onset from one representative embryo for each genotype. Scale as indicated, gray indicates periods where nuclei were not tracked, n= 68, 70, 60 nuclei for *ush* and 61, 65, 28 nuclei for *hnt>ush*. Time of transcriptional onset was traced to visualize onset fronts of different regions in *ush* WT embryos and superimposed onto the same regions in *hnt>ush* embryos. The time at which half the nuclei in a region initiated transcription is indicated as T_{50} (25.2, 27.5 and 33.5 min for *ush* and 23.9, 29.1, 34.3 min *hnt>ush*).

(Ciii) The mean number of elongating Pol II molecules was calculated for each biological replicate based on the BMP signaling regions (n=3 embryos).

Mean ± 95% confidence intervals (B), mean ± SD (Ciii); ns, not significant. Student’s test (Ciii).

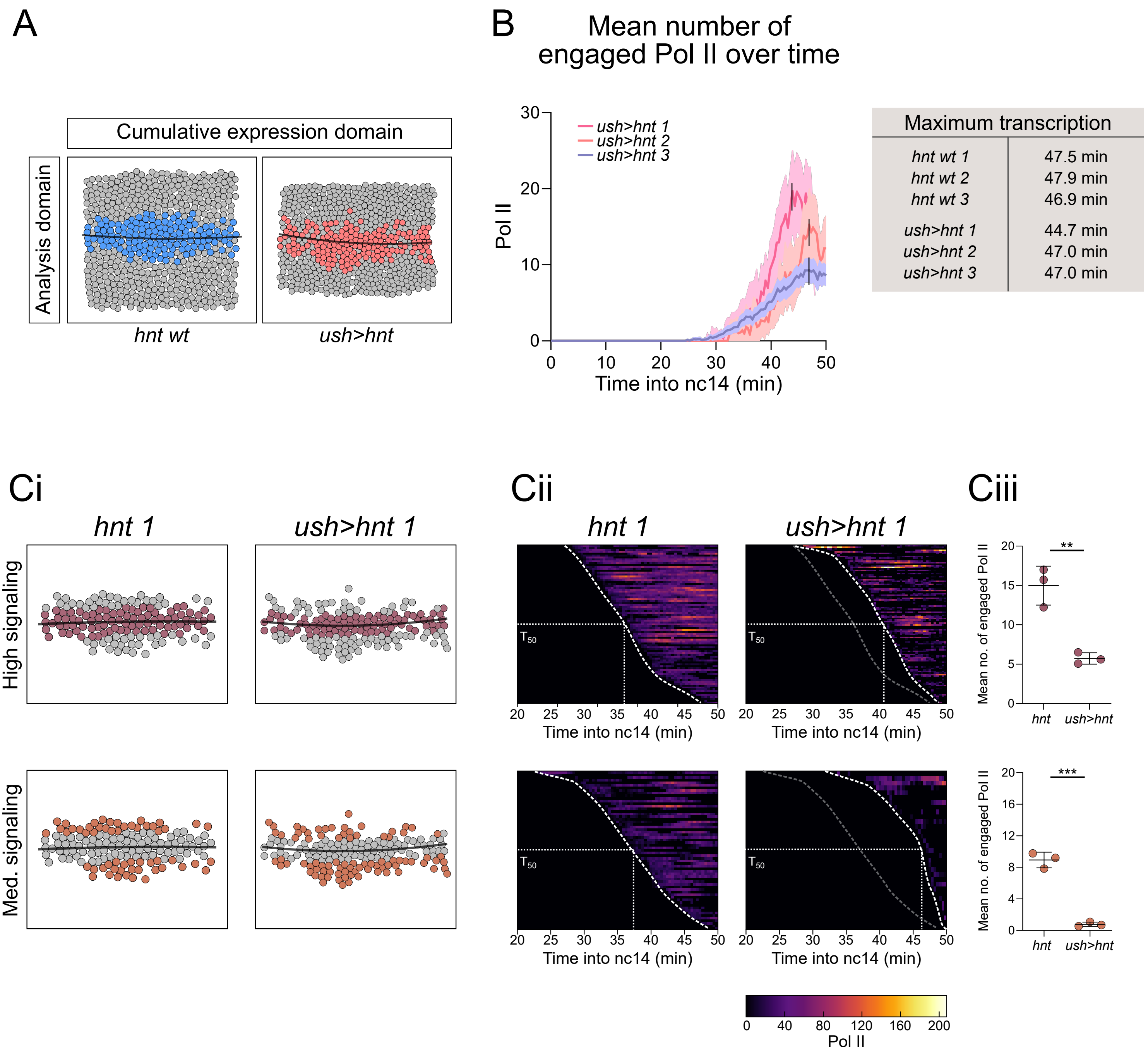
A
Cumulative expression domain
Analysis domain
hnt wt
ush>hnt
B
Mean number of engaged Pol II over time
ush>hnt 1
ush>hnt 2
ush>hnt 3
Pol II
Time into nc14 (min)
Maximum transcription
hnt wt 1 47.5 min
hnt wt 2 47.9 min
hnt wt 3 46.9 min
ush>hnt 1 44.7 min
ush>hnt 2 47.0 min
ush>hnt 3 47.0 min
Ci
hnt 1
ush>hnt 1
High signaling
Med. signaling
Cii
hnt 1
ush>hnt 1
T50
Time into nc14 (min)
Pol II
Ciii
Mean no. of engaged Pol II
**
***
hnt
ush>hnt

Figure S7: Analysis of *ush>hnt* embryos. Related to Figure 7.

(A) The cumulative *hnt* expression domain in *hnt* (blue) and *ush>hnt* (pink) embryos.

(B) Mean *hnt* transcription over time in *ush>hnt* embryos, with the time of maximum transcription listed for all biological replicates including *hnt* MS2 WT embryos for comparison (n= 3, 133, 187 and 202 nuclei).

(Ci) Schematics of representative embryos showing the analysis regions.

(Cii) Heatmaps of single-cell traces, sorted according to transcription onset (scale as indicated, n= 83, 61 nuclei for *hnt* and 71, 56 nuclei for *ush>hnt*) from one representative embryo for each genotype. The transcriptional onset front was traced in *hnt* WT embryos and superimposed onto the same regions in *ush>hnt* embryos. The T_{50} times are as follows: 39 and 39.2 min for *hnt* and 43 and 38.3 min for *ush>hnt*.

(Ciii) The mean Pol II number was calculated for each biological replicate based on BMP signaling regions (n=3 embryos) (iii).

Mean ± 95% confidence intervals (B), mean ± SD (Ciii). $^{**}p < 0.01$, $^{***}p < 0.001$. Student’s test (Ciii).

Table S1: smFISH probes used for FISH and complementary to exonic sequences in *hnt* and *ush,* related to STAR Methods.

Gene	Probe	sequence	Gene	Probe	sequence
hnt	1	aatggcgaattttgcgcttg	*hnt*	25	cggaatagctgctgcatata
hnt	2	tagtccatcacaatggatgc	*hnt*	26	gtctgggactggaacatgag
hnt	3	gatagcaccttggagcaaat	*hnt*	27	ggtggtgccataaagggaaa
hnt	4	gcaacttgatgctgtttgtg	*hnt*	28	gattgatacggtttggtgga
hnt	5	agattgttgttagccttgtg	*hnt*	29	aggactccattcttgatgac
hnt	6	atgttgttgttgatggtggt	*hnt*	30	agcagtgttcacaggcaaag
hnt	7	ccaaaagagtggcgacatcg	*hnt*	31	cgaacgcagcgtaaatctcg
hnt	8	gacaacttaggagcaaggca	*hnt*	32	ttgtgcatagaactgcggat
hnt	9	tggtatgtctgagtgcattg	*hnt*	33	cgaaatgggagcatgaccat
hnt	10	gacgatgggtggcaaagatc	*hnt*	34	caacagatcggtgttcttgt
hnt	11	gtaagcgacaacagtgcgac	*hnt*	35	ccaaagtgcaggagaggatt
hnt	12	tgtcttgtgcttcagataca	*hnt*	36	ctcatgatcatgctcgtttt
hnt	13	ttcaggatgctctggatatc	*hnt*	37	ggctgctcatctagaatcaa
hnt	14	aaactggaactggagctggt	*hnt*	38	cttgagcgatcatcgttttc
hnt	15	ttcacggactgctcaaagtt	*hnt*	39	acttctttgagggattttcc
hnt	16	tgtactgactcgaattgggc	*hnt*	40	atccttgaacggtgaactgg
hnt	17	ggcacgaaggttcgggaaaa	*hnt*	41	tggcaatacggacaggagac
hnt	18	aaatgtacccgattgtgacc	*hnt*	42	tcttggtggtgaacaacagc
hnt	19	aaacggcgtagggacacatg	*hnt*	43	cagcgactcagtttcaatgg
hnt	20	tgaaggcatagttgcacacg	*hnt*	44	gcaagcagaaggcacagata
hnt	21	ctcagatgacgttcacagtt	*hnt*	45	tgtgcttcagtgtgaacttc
hnt	22	gtgggactgatcgaacggaa	*hnt*	46	tcactgatgcccaatagatt
hnt	23	ctgggatttgctctcattta	*hnt*	47	tactccagtatttatggctc
hnt	24	gctgagatccaaaacatcca	*hnt*	48	ctttggtatacgtaaggcgg
ush	1	tctgcggtatcggaacaatc	*ush*	25	gtccagggaagtgtacttaa
ush	2	atctttggaatctctgctgt	*ush*	26	ggggcaatagtagttctgat
ush	3	ggaatcgttcaactgatcct	*ush*	27	ggacaaggcagaccaatttc
ush	4	cgaactcagcatcttcatca	*ush*	28	acaggacacttgttcagact
ush	5	tgaagatcgtgttcctgttc	*ush*	29	aatgggttcgaatgtgggtg
ush	6	acagggcaggcacataaatc	*ush*	30	agatgcaggtcatgtatagc
ush	7	ttgagggcgaactgaatgca	*ush*	31	agggatgatccatggaatcg
ush	8	tccgtgtccttaattctatg	*ush*	32	aggtggagacatagttgcag
ush	9	catgtggcgatttagggata	*ush*	33	agtcacatcttgatctctgg
ush	10	ctattccgttctggagaaga	*ush*	34	accgttggatacatcggaat
ush	11	cggtttgttgactagagcta	*ush*	35	gagcagtacttcttcatcac
ush	12	cttgatgttgttgaaccgga	*ush*	36	caggtaggtcttcacgtaat
ush	13	cgaactgcagtagtgttgtt	*ush*	37	tgcagtagaactgcttgtga
ush	14	aaaggtggatgcgaatgcgg	*ush*	38	cgtgtaataccactcaagct
ush	15	acaattatgatcgggtgggt	*ush*	39	ggaggctaggattcgattag

Gene	Probe	sequence	Gene	Probe	sequence
ush	16	tgggcctggaataaagctag	*ush*	40	ttgcggtttggatagtgtac
ush	17	cagattctctagttaaccct	*ush*	41	ggagattttccgggaatact
ush	18	aagctcagtgaattcagggt	*ush*	42	tcggtaagtcgaaggagtca
ush	19	cttccacatctaagagagca	*ush*	43	tgtgttattcctttaggtgt
ush	20	gagggagcgcaaacgatttg	*ush*	44	gtaagctttggcatgcatta
ush	21	actactagtttgggcaggaa	*ush*	45	gccctcaatttaatttctgt
ush	22	cccttttcacatagatctg	*ush*	46	cgcagaccattgcaaacttg
ush	23	acaatattgcactccatgca	*ush*	47	agaattgctcgctttatggg
ush	24	tgccaagtagttctcatact	*ush*	48	cttttattgtcgcacacact
